# Antibody-Functionalized Polymer Nanoparticles for Targeted Antibiotic Delivery in Models of Pathogenic Bacteria Infecting Human Macrophages

**DOI:** 10.1021/acsami.3c07367

**Published:** 2023-08-19

**Authors:** Laura Gabriela Miranda Calderon, Teresa Alejo, Sabas Santos, Gracia Mendoza, Silvia Irusta, Manuel Arruebo

**Affiliations:** † Instituto de Nanociencia y Materiales de Aragón (INMA), CSIC-Universidad de Zaragoza, Zaragoza 50009, Spain; ‡ Department of Chemical Engineering, University of Zaragoza, Campus Río Ebro-Edificio I+D, C/ Poeta Mariano Esquillor S/N, Zaragoza 50018, Spain; § Networking Research Center on Bioengineering, Biomaterials and Nanomedicine, CIBER-BBN, Madrid 28029, Spain; ∥ Aragon Health Research Institute (IIS Aragon), Zaragoza 50009, Spain

**Keywords:** infection, antibiotic, antibody-functionalized nanoparticles, PLGA, biofilm, Staphylococcus aureus

## Abstract

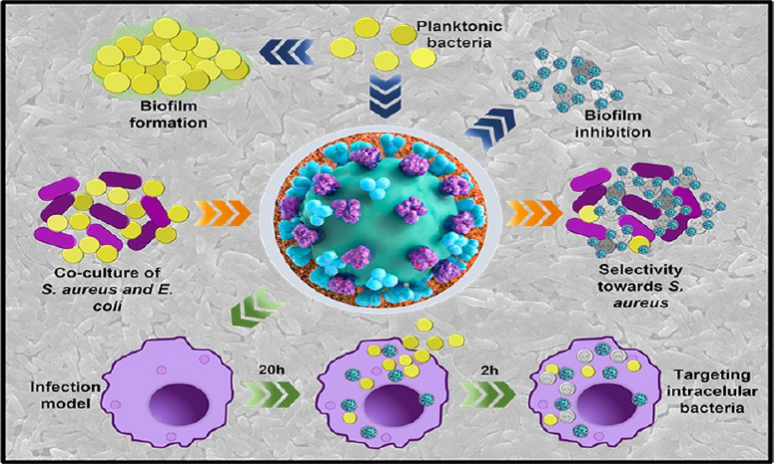

The efficacy of antibody-functionalized poly(d,l-lactide-co-glycolide) (PLGA) nanoparticles (NPs), prepared by nanoprecipitation, carrying rifampicin (RIF) against planktonic, sessile, and intracellular *Staphylococcus aureus* and *Escherichia coli* is reported here. A biotinylated anti-*S. aureus* polyclonal antibody, which binds to structural antigens of the whole bacterium, was functionalized on the surface of RIF-loaded PLGA-based NPs by using the high-affinity avidin–biotin complex. This general strategy allows the binding of commercially available biotinylated antibodies. Coculture models of *S. aureus* ATCC 25923 and *Escherichia coli* S17 were used to demonstrate the preferential selectivity of the antibody-functionalized NPs against the Gram-positive bacterium only. At 0.2 μg/mL, complete *S. aureus* eradication was observed for the antibody-functionalized RIF-loaded NPs, whereas only a 5-log reduction was observed for the nontargeted RIF-loaded NPs. *S. aureus* is a commensal facultative pathogen having part of its live cycle intracellularly in both phagocytic and nonphagocytic cells. Those intracellular bacterial persisters, named small colony variants, have been postulated as reservoirs of relapsed episodes of infection and consequent treatment failure. At 0.5 μg/mL, the RIF-loaded NPs reduced in 2-log intracellular *S. aureus*-infecting human macrophages. The ability of those antibody-functionalized nanoparticles to prevent biofilm formation or to reduce the bacterial burden in already-formed mature biofilms is also reported here using *S. aureus* and *E. coli* single and cocultured biofilms. In the prevention of *S. aureus* biofilm formation, the antibody-functionalized NPs exerted a superior inhibition of bacterial growth (up to 2 logs) compared to the nonfunctionalized ones. This study demonstrates the selectivity of the synthesized immunonanoparticles and their antimicrobial efficacy in different scenarios, including planktonic cultures, sessile conditions, and even against intracellular infective pathogens.

## 1 Introduction

Antibiotic selectivity toward bacteria is achieved by targeting specific bacterium receptors or by interfering with biomolecular processes exclusive to prokaryotes. Despite their high efficacy, bacteria have developed resistance to the antibiotic selective pressure by using different counteracting mechanisms, including the increase in the activity of their efflux pumps, direct antibiotic inactivation, and reduction in the antibiotic binding affinity, by modifying the bacterial target, by reducing the outer membrane permeability, replacing or bypassing the original target, and so on.^1^ As a consequence, commonly used antibiotics are becoming progressively ineffective while multi- and pan-resistant bacteria rapidly spread around the globe.^2^

Nanomaterials have greatly contributed to major advances in antimicrobial therapy by increasing the potency or bioavailability of existing antibiotics or by their inherent mechanisms of antimicrobial action, such as in the case of metal nanoparticles.^3^ In addition, several of the nanomaterials used in antimicrobial therapy show multiple mechanisms of antimicrobial action, and this lack of target specificity leads to a reduction in the probability of developing resistance. As carriers of therapeutic antimicrobials, nanoparticles can increase the therapeutic index by delivering the cargo in close proximity to the pathogenic bacteria by using targeting surface moieties. The affinity of those targeting biomolecules toward the receptor overexpressed on the surface of the bacterial cell is responsible for a superior antimicrobial action of surface-functionalized drug-loaded polymer nanoparticles in comparison to the effect of equivalent doses of the corresponding transported free drug. The selectivity toward bacterial cells has been achieved by using different natural and synthetic targeting biomolecules, including peptides, aptamers, carbohydrates, cell membranes, monoclonal, polyclonal, and recombinant antibodies.^4^ This selectivity has been explored in the identification and diagnosis of specific pathogenic bacterial strains or to increase the therapeutic efficacy of antimicrobial treatments.

One of the common commensal bacteria that can become pathogenic is the opportunistic *Staphylococcus aureus*. Implant-associated infection, endocarditis, skin and soft tissue infection, pneumonia, osteomyelitis, and even bacteremia are common clinical manifestations of its virulence.^5^ Selective antibody-functionalized nanoparticles against epitopes of *S. aureus* have been developed to detect its presence. For instance, immunomagnetic capture and subsequent surface-enhanced Raman scattering (SERS) detection using Au-coated magnetic nanoparticles in bacterial suspensions has been reported using monoclonal antibodies as targeting moieties.^6^ Immunomagnetic nanoparticles have also been used to capture and concentrate methicillin-resistant *S. aureus* (MRSA) from human nasal swabs using a microfluidic device, and subsequently, the strain was identified using an antibody-functionalized with specific enzymes for its electrochemical detection.^7^ Simultaneous detection and antimicrobial treatment have been widely described when using theragnostic nanoparticles. For instance, Huo et al.^8^ described the functionalization of Au/Ag nanoparticles with anti-MRSA monoclonal antibodies and their use as contrast agents for computed tomography (CT) in ventilator-associated MRSA pneumonia murine models, showing, in addition, an efficient bacterial proliferation inhibition in vivo. Anti-protein A antibody-functionalized nanoparticles have been used for the selective elimination of pathogenic *S. aureus* by nanoparticle-assisted magnetic fluid hyperthermia in the management of infected nonhealing wounds^9^ or by using metal nanoparticles when applying photothermal therapy alone or in combination with antibiotics.^10^ Antibody anti-MRSA-functionalized metal nanoparticles conjugated with photosensitizers were also used in photodynamic therapy to increase the selectivity toward bacteria when cultured alongside eukaryotic cells.^11^ Not only metal nanoparticles were used to selectively reduce bacterial infection, but also inorganic systems, such as vancomycin-loaded porous silicon nanoparticles functionalized with a cyclic 9-amino-acid peptide, have shown improved antibacterial bioavailability and selectivity against *S. aureus* in vivo.^12,13^ Also, polymeric nanoparticles have been used to selectively deliver antibiotics against *S. aureus*; for example, nanoparticles based on poly-(d,l-lactide-co-glycolic acid) (PLGA) and polyethylene glycol (PEG) were loaded with rifampicin and surface functionalized with the anti-protein A antibody, used as a targeting ligand, showing improved therapeutic efficacy in a murine infection model created by implanting biofilm-containing grafts subcutaneously.^13^ Compared to metal or inorganic nanoparticles, polymeric ones release their encapsulated antimicrobial in a controlled and sustained manner, they show tunable physical and chemical properties which allow endogenous (i.e., enzymatic, hydrolytic, pH or glutathione-responsive, etc.) or exogenous (i.e., activated by light, magnetic, ultrasound-responsive, etc.) biodegradation, and they show design flexibility based on their easy surface functionalization, the availability of many different natural and synthetic polymers, and varied macromolecular synthesis methods.

However, the lack of studies on polymeric nanoparticles highlights the need for further investigation in this area, presenting an opportunity for an extensive exploration of targeted applications.

In summary, stand-alone antibodies (e.g., Panobacumab, Tefibazumab, etc.), antibody-antibiotic conjugates,^14^ and antibody-functionalized nanoparticles^12,13^ have been successfully used in the treatment of bacterial infections taking advantage of their biological selectivity against unique bacterial epitopes. However, some pathogenic bacteria remain part of their life cycle intracellularly in phagocytic cells. In those cases, the pathogen remains in the endosomal–lysosomal system, and the recognition ability of the antibody is hindered. For instance, *S. aureus* is a commensal facultative pathogen spending part of its live cycle intracellularly.^15^*S. aureus* also infects nonphagocytic cells, with their intracellular persistence being attributed to small colony variants.^16^ Those intracellular bacterial persisters have been postulated as reservoirs of relapsed episodes of infection and consequent treatment failure.^17^ In addition, respiratory, periodontal, urinary, skin, and soft tissue infections are polymicrobial in nature,^18,19^ and consequently, antibiotic or antiseptic treatments should consider microbial community interactions of pathogenic and commensal bacteria as well as all of those pathogens living part of their life cycle intracellularly.

In the current study, we have analyzed the efficacy and selectivity of antibody-functionalized nanoparticles carrying antibiotics against planktonic, sessile, and intracellular *S. aureus* in order to assess the efficacy of the targeted antibiotic therapy in different settings. In addition, coculture models of *Escherichia coli* and *S. aureus* have been used to demonstrate the selectivity of the targeting moiety selected. Additionally, a model of infected human macrophages has also been used to demonstrate the ability to target intracellular persisters. To the best of our knowledge, this is the first time that the selectivity of antibody-functionalized antibiotic-loaded nanoparticles has been evaluated in the same study in competitive models of different bacteria in coculture in both planktonic and sessile forms, in infection models of intracellular bacteria alone or in a combination of two bacteria, and in the prevention and inhibition of biofilm formation. The cytotoxicity of those immunonanoparticles is also reported here. We are aware of the fact that rifampicin is always used in combination with other antibiotics, and monotherapy is not recommended against biofilm-forming bacteria due to its high chances of developing resistance;^20,21^ however, we have chosen it as a model antibiotic to just analyze the efficacy of targeted nanoparticles compared to standard nontargeted ones. In potential future applications, combination therapies are envisaged. Herein, PLGA nanoparticles have been used to take advantage of their physiological biodegradability by hydrolysis of its ester bonds, augmented cellular uptake by endocytosis, drug protection, enhanced drug stability, and controlled release ability. Importantly, these results demonstrate their potential to effectively inhibit or even eradicate *S. aureus*-associated infections, even in the most challenging scenarios.

## 2 Experimental Section

### 2.1 Materials

Dimethyl sulfoxide (DMSO) > 99%, phosphate-buffered saline (PBS), acetone (ACS reagent, ≥99.5%), rifampicin (RIF) ≥ 97%, chloroform-*d* (99.8 atom % D), diethyl ether (99.7%), methanol (99.8%), chloroform (99%), *N*-(3-dimethylaminopropyl)-*N*’-ethylcarbodiimide hydrochloride (EDC), avidin from egg white (Millipore), *N*,*N*-diisopropylethylamine (DIEA, > 99.5%), *N*-hydroxysuccinimide (NHS, 98%), and dichloromethane (99.8%) were purchased from Sigma-Aldrich (Darmstadt, Germany). Resomer RG 503 H was purchased from Evonik Industries GmbH. *S. aureus* polyclonal antibody–biotin was purchased from Thermo Fisher (Waltham, Massachusetts). Biotin–PEG3400–NH_2_ was purchased from Xi’an ruixi Biological Technology Co., China. All mentioned chemicals were used as received. Tryptone soy broth (TSB) and tryptone soy agar (TSA) were acquired from Laboratorios Conda-Pronadisa S.A., Madrid, Spain. *S. aureus* ATCC 25923 was acquired from Ielab (Alicante, Spain), and *Escherichia coli* S17 was a generous gift from Dr. Jose A. Ainsa, University of Zaragoza (Zaragoza, Spain).

### 2.2 Synthesis of the PLGA–PEG–Biotin Copolymer

To prepare the copolymer, 1 g of the acid-terminated PLGA (PLGA-COOH, Resomer RG 503 H, 20 kDa MW) was dissolved in dichloromethane (4 mL) by stirring at 25 °C in the presence of NHS (1:8 PLGA/NHS molar ratio) and EDC (1:8 PLGA/EDC molar ratio) to form an amine-reactive ester, which was subsequently conjugated with the biotinylated PEG–NH_2_. The excess of NHS and EDC was eliminated using a solution containing 70/30 vol % ethyl ether and methanol, and, after washing, a vacuum was applied for 4 h to remove any remaining solvent leftovers. For characterization, 10 mg of the resulting PLGA–NHS was collected and stored at −20 °C for proton nuclear magnetic resonance (H-NMR) analysis. The polymer was then dissolved again in 5 mL of chloroform previously purged with argon and contacted under moderate stirring with NH_2_–PEG–biotin (3400 MW, 1:1.3 PLGA/PEG molar ratio) and 2 mL of DIEA overnight. Methanol was used to wash the resulting polymer to eliminate unreacted PEG. The resulting PLGA–PEG–biotin was recuperated using ethyl ether, vacuum dried for 2 h, and stored at −20 °C. For chemical characterization, 10 mg of PLGA–PEG–biotin was collected and stored at −20 °C for H-NMR analysis following a previously reported protocol.^22^ Samples stored for H-NMR analysis were each dissolved in chloroform-*d* to reach a concentration of 10 mg/mL and loaded in glass H-NMR tubes. Samples were measured in a Bruker Advance 400 Mhz NMR spectrometer to verify the effective PEG conjugation to PLGA and to evaluate the possible presence of intermediary products.

### 2.3 Synthesis of PLGA–PEG–Biotin Nanoparticles by Nanoprecipitation

In the first step, precursor solutions were prepared as follows: a solution of 10 mg/mL of PLGA–PEG–biotin was prepared in acetone. Also, using acetone as a solvent, a 5 mg/mL solution of RIF was prepared. The working solution was obtained by mixing PLGA–PEG–biotin solution 50% v/v and RIF solution 20% v/v in a final volume of 1 mL. This solution was then mixed with 1 mL of ultrapurified water under stirring (300 rpm) using a Harvard Apparatus Standard PHD Ultra syringe pump at 2 mL/h flow rate. The collected solution was stirred (300 rpm) at 25 °C for 2 h to allow polymer precipitation and solvent evaporation. The resulting nanoparticles (NPs) were collected by ultrafiltration (5500 rpm) during 5 min (EBA21 centrifuge, Hettich, Tuttlingen, Germany) using an Amicon Ultra-4, 100 kDa centrifugal filter (Millipore). NPs were redispersed in 0.3 mL of ultrapurified water and kept at 4 °C.

### 2.4 Anti-*S. aureus* Antibody Conjugation

**Figure 1:**
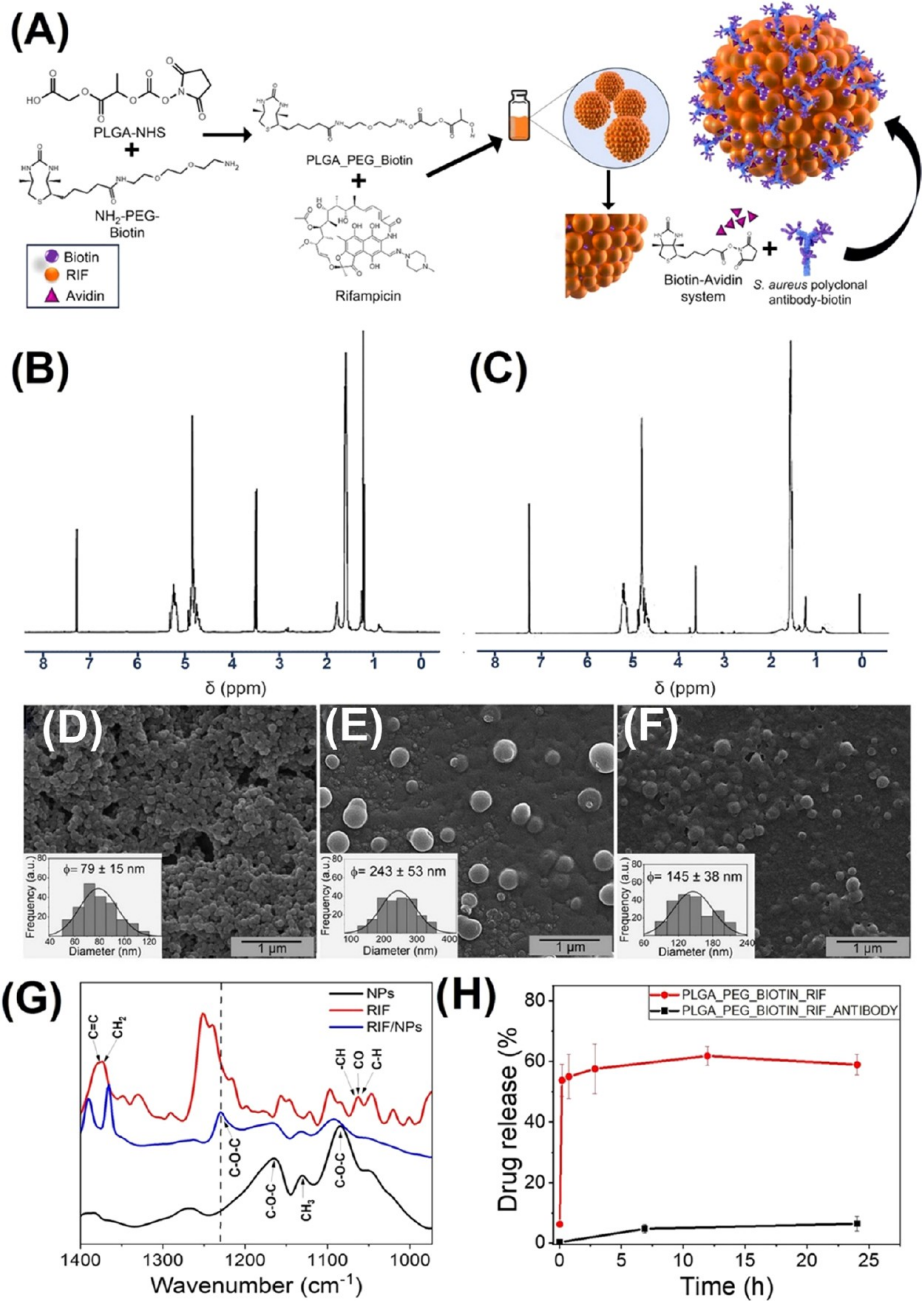
(A) Schematic description of the conjugated system formed by surface functionalizing PLGA–PEG–biotin nanoparticles with the biotinylated anti-*S. aureus* antibody. Nanoparticle characterization: (B) H-NMR spectra of the incorporation of NHS to the acid-terminated PLGA polymer. (C) H-NMR spectra of the conjugation of the modified PLGA polymer to NH_2_–PEG–biotin. (D) The SEM image and size distribution histogram of PLGA–PEG–biotin NPs. (E) The SEM micrograph and size distribution histogram of PLGA–PEG–biotin–avidin–RIF NPs. (F) The SEM micrograph and size distribution histogram of PLGA–PEG–biotin–avidin–RIF–antibody NPs. (G) FTIR spectra of RIF, unloaded NPs, and RIF-loaded NPs. (H) RIF release profile from NPs containing the antibody or not (*n* = 3). The size of the NPs is representative (*n* ≥ 100; mean ± SD).

The schematic description of the conjugated system formed by surface functionalizing PLGA–PEG–biotin nanoparticles with the biotinylated anti-*S. aureus* antibody is shown in Figure 1A. The biotinylated antibody was conjugated to the NPs using the avidin–biotin system.^23^ First of all, avidin was conjugated to the NPs functionalized with biotin. For avidin conjugation, 500 mL of NP dispersion (20 mg/mL) were incubated with 2 mL of avidin solution (2 mg/mL) in a closed flask and gently rotated on a roller shaker for 30 min at 4 °C to conjugate avidin with the nanoparticles. The free avidin-binding protein was eliminated by ultrafiltration at 5500 rpm for 5 min using an Amicon filter. The final nanoparticles were resuspended using 0.3 mL of DDI water. Then, 150 μL (20 mg/mL) of avidin-modified nanoparticles and 2.8 μL (4.5 mg/mL) of biotinylated anti-*S. aureus* polyclonal antibodies were thoroughly mixed and incubated at 4 °C for 15 min. These amounts were selected after performing an optimization of the binding ability of the NPs with different amounts of biotinylated antibody (results not shown). Samples were stored at 4 °C for subsequent analysis.

### 2.5 Nanoparticle Characterization

Electrophoretic light scattering (ELS) was used to calculate the ζ potential values of colloidal suspensions of the NPs using a Brookhaven 90 plus particle size analyzer (Holtsville, New York) in 1 mM aqueous KCl solution at pH = 6.5. Nanoparticle hydrodynamic sizes were determined by dynamic light scattering (DLS) using the same piece of equipment operating at a detection angle of 90° in an aqueous suspension. Scanning electron microscopy (SEM) was used to visualize the morphology of the obtained NPs. To make the samples electron conductive, a thin layer of Pd was sputtered on the samples, and images were acquired using an Inspect F50 FEG scanning electron microscope (FEI Company, Hillsboro, Oregon). NP sizes were measured (*N* = 100) using ImageJ Software (Version Java 1.8.0_172).

RIF encapsulation efficiency (EE), drug loading (DL), and release profile of RIF-loaded PLGA–PEG–biotin NPs were analyzed using a V-670 UV–VIS–NIR Jasco (Jasco Applied Science, Eschborn, Germany) spectrophotometer. Samples were prepared by dissolving 0.1 mL of antibiotic-loaded PLGA–PEG–biotin NPs in 0.9 mL of DMSO.

The encapsulation efficiency (EE) and drug loading (DL) were estimated using eqs 1 and 2
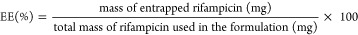
1
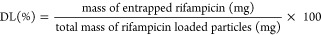
2

Chemical interactions between the NP components were studied by Fourier transform infrared spectroscopy (FTIR) using an FTIR Vertex-70 (Bruker, Billerica, Massachusetts), having a Golden Gate ATR accessory. The presence of avidin and the antibody was determined indirectly using the Pierce BCA Protein Assay Kit (ThermoFisher, Waltham, Massachusetts) using the unbound avidin and antibody collected from the centrifugal washes and performing a mass balance. Standards of different concentrations were prepared ranging from 0 to 2000 μg/mL, working reagent for standards, and unknown samples were prepared by mixing BCA reagent A with BCA reagent B (50:1 ratio). Then, 25 μL of each standard and unknown sample were mixed with 200 μL of working reagent in a microplate well (Thermo Scientific Pierce 96-well plate, ThermoFisher, Waltham, Massachusetts) and mixed on a plate shaker for 30 s. The plate was protected with aluminum foil and incubated at 37 °C for 30 min. After cooling down the plate to RT, the absorbance was measured at 562 nm on a Synergy HT Multi-Detection Microplate Reader (BioTek Instruments, Agilent Technologies, Santa Clara, California).

### 2.6 Antibacterial Activity

Using *S. aureus* ATCC 25923 as a model of a Gram-positive bacteria and using *E. coli* S17 strain as a model of a Gram-negative bacteria placed in 96-well microplates, we calculated the minimal inhibitory concentration (MIC) and the minimum bactericidal concentration (MBC) using the standard microdilution method for the free antibiotic (RIF). To do so, both microorganisms were grown in TSB at 37 °C under continuous shaking (150 rpm) overnight until the stationary growth phase (10^9^ CFU/mL) was reached; then, the cultures were diluted in TSB until reaching 10^5^ CFU/mL. The resulting inoculum was placed into test tubes containing a known quantity of RIF (0–0.5 μg/mL for *S. aureus* and 0–60 μg/mL for *E. coli*) dissolved in 2% DMSO. After 24 h of incubation at 37 °C and 150 rpm stirring rate, the standard serial dilution method was employed to quantify viable bacteria. As a positive control, untreated *S. aureus* and *E. coli* S17, respectively, were also included, and a toxicity control using DMSO only was also performed in parallel again, and each experiment was performed in triplicate.

The same process described above to determine MIC and MBC values was performed to analyze the antimicrobial effect of the RIF-loaded NPs with and without the surface targeting antibody. A bacterial inoculum of 10^5^ CFU/mL was incubated with varied NP concentrations (0.1–2 μg/mL) for 24 h, and the microdilution method was carried out again. Each experiment was performed in triplicate using two replicas for each sample from different syntheses. Nanoparticles were sterilized under UV light for 30 min prior to being used in the experiment. The potential UV degradation of RIF was studied using a Synergy HT Multi-Detection Microplate Reader (BioTek Instruments, Agilent Technologies, Santa Clara, California). A 10 μg/mL solution of RIF dissolved in DMSO was placed in a 96-well microplate, and the absorbance at 334 nm was read prior to the sterilization process and after 30 min of UV light exposure following previously reported protocols.^24^

### 2.7 Coculture Antibacterial Activity on Prokaryotic Cells

A standard suspension of *S. aureus* and *E. coli* was prepared from a 16 h culture grown in TSB at 37 °C. Each culture was diluted to reach 10^7^ CFU/mL, then the same volume of each bacteria was placed in the same well along with nanoparticles of RIF-loaded PLGA–PEG and antibody-functionalized RIF-loaded PLGA–PEG NPs in concentrations varying from 0.2 to 2 μg/mL for 24 h. After that, the microdilution method was carried out as it has been mentioned above.

### 2.8 Antibiofilm Activity

The effects of PLGA–PEG–biotin NPs with and without the targeting antibody in the prevention of biofilm formation and their capacity to disrupt already-formed mature biofilms of *S. aureus*, *E. coli*, and cocultures of both bacterial biofilms were also studied. To evaluate the effect on the prevention of biofilm formation, nanoparticles (0.5–3 μg/mL) were added to bacteria (10^7^ CFU/mL), and samples were incubated for 24 h at 37 °C without stirring. In the case of the coculture, the same amount of both bacteria was added to reach a final concentration of 10^7^ CFU/mL. After incubation, biofilms were washed and disrupted by using a sonication probe (15 min, 200 W; Ultrasons, JP Selecta, Barcelona, Spain), subsequently diluted and seeded in agar plates; after 24 h of incubation at 37 °C, viable colonies were counted. For the cocultured biofilm, selective media were employed; Columbia CAN supplemented with 5% sheep blood was used for *S. aureus* biofilms, whereas MacConkey agar was employed for *E. coli* biofilms.

To evaluate the disruption of preformed biofilms, bacteria were inoculated (10^7^ CFU/mL) in a 96-well microplate for 24 h at 37 °C without stirring as described before, then NPs in a concentration of 0.5–2 μg/mL were added and incubated for 24 h at 37 °C without stirring. After incubation, free planktonic colonies were washed twice with PBS, and the biofilm samples were disrupted by using a sonication probe and seeded as described above. After 24 h of incubation at 37 °C, the number of viable bacterial colonies remaining (CFU/mL) was counted.

Moreover, to observe the effect that PLGA–PEG–biotin NPs with and without antibodies displayed on biofilm formation prevention and on biofilm disruption in mature biofilms, two methodologies were carried out:

- SEM: Bacteria were grown for biofilm formation prevention and disruption analyses and treated with NPs as before in 24-well plates with a glass slide at the bottom. After treatment, each slide was washed with PBS and immersed in 500 μL of PFA overnight. Then, the samples were rinsed with distilled water and 70% ethanol and left to air-dry. A thin Pt coating was used to make the samples electron conductive, and those were visualized using a scanning electron microscope (SEM) Inspect F50 (FEI Co., Hillsboro, Oregon).
- Confocal microscopy: *S. aureus*, *E. coli*, and cocultures of both bacteria were grown onto poly-l-lysine-coated μ-slide eight-well glass-bottom plates (Ibidi, Germany) for biofilm formation and biofilm disruption analyses. Samples were treated with antibiotic-loaded NPs with and without targeting antibodies for 24 h, as described before. Then, the culture medium was eliminated, and the biofilms were rinsed with PBS to remove nonadherent bacteria. Subsequently, 300 μL of 4% PFA in PBS were added to each well. After 45 min, PFA was washed twice with sterile water, and 200 μL of 1.67 μM of SYTO 9 were added and incubated for 30 min. Then, each well was washed with sterile water and stained with 200 μL of 0.025% Calcofluor White stain for 30 min to be later envisioned by confocal microscopy (Confocal Zeiss LSM 880 with Airyscan, Zeiss, Jena, Germany). Untreated biofilms were also used as control.

### 2.9 Cytotoxicity Study

The evaluation of the cytotoxicity of the reported NPs with and without targeting antibodies was assayed in J774 macrophage cultures. Dulbecco’s modified Eagle’s medium with high glucose (DMEM; Biowest, Nuaillé, France) supplemented with 10% fetal bovine serum (FBS; Thermo Fisher Scientific, Waltham, Massachusetts) and 1% penicillin–streptomycin–amphotericin B (PSA; Biowest, Nuaillé, France) was used to grow the cells by incubating them at 37 °C in a 5% CO_2_ atmosphere. Macrophage viability after treatment with RIF-loaded PLGA–PEG NPs functionalized with and without the targeting antibody at concentrations between 0.2 and 2 μg/mL was analyzed by the Blue Cell Viability Assay Kit (Abnova, Taipei, Taiwan).

Cells were seeded in 96-well microplates (18 000 cells/cm^2^) and incubated with both kinds of NPs for 24 h. Then, the manufacturer’s instructions (10%; incubation of 4 h at 37 °C and 5% CO_2_) were followed to analyze the fluorescence displayed after incubating the cells with the kit reagent using a multimode microplate reader (Varioskan LUX; Thermo Fisher Scientific, Waltham, Massachusetts) at 530:590 nm (excitation/emission) wavelengths. Viability was calculated by interpolating the fluorescence data from the cells treated with RIF-loaded PLGA–PEG NPs functionalized with and without the targeting antibody versus the nontreated cells (control sample, assigned with 100% viability). The experiments were performed in triplicate.

### 2.10 Evaluation of the Nanoparticle’s Antibacterial Activity in the Infection Model

To test the efficacy of the NPs with and without the targeting antibody against an intracellular infection mediated by *S. aureus* or *E. coli* or by both bacteria, a previously reported protocol was carried out.^25^ Briefly, RIF-loaded PLGA–PEG NPs with and without the targeting antibody were added to the seeded cells in 24-well microplates (18 000 cells/cm^2^) at the bactericidal concentration for the antibody-functionalized NPs previously determined (0.2 μg/mL) 20 h before infection. Then, macrophages were infected using *S. aureus* at a multiplicity of infection (MOI) of 20:1, while *E. coli* infection was generated using an MOI of 8:1. Cocultures of *S. aureus* and *E. coli* were also tested by the addition of both bacteria at the same MOIs used separately (20:8:1). Control samples were also prepared as physiological control (not treated and not infected) and as infection control (not treated and infected). After infection, plates were centrifuged at 200*g* for 5 min and incubated for 30 min at 37 °C. Later, cells were washed twice with PBS and treated with a solution of 100 μg/mL gentamicin sulfate for 1 h at 37 °C in order to eradicate noninternalized bacteria. Then, to break the cell membrane and retrieve intracellular bacteria, cells were washed twice with PBS and treated with 250 μL of Triton X-100 (0.5%) for 15 min. The final suspensions were diluted in PBS and seeded following the conventional microdilution method on TSA.

Moreover, the viability of macrophages after infection was evaluated by using confocal microscopy by the Live/Dead Viability/Cytotoxicity Kit for mammalian cells (Thermo Fisher Scientific, Waltham, Massachusetts). Viability tests were performed to ensure that the appropriate MOIs were used in the infection model with the NPs reported. Cells were seeded in a 12-well plate and then infected as described above. Afterward, cells were washed twice with PBS, and we added a solution containing 20 μL of 2 mM ethidium homodimer-1 (EthD) stock solution and 5 μL of 4 mM calcein AM. After 15 min of incubation at 37 °C, samples were analyzed by confocal microscopy (Leica TCS SP2 Laser Scanning Confocal Microscope, Wetzlar, Germany).

### 2.11 Statistical Analyses

All results reported in this work were calculated as mean ± standard deviation (SD). We used the two-way analysis of variance (ANOVA) to statistically analyze the cellular experimental results (GraphPad Prism 9, San Diego). We considered statistically significant differences when *p* ≤ 0.05. The nanoparticle characterization experiments were conducted in quadruplicate, whereas the biological analysis experiments were carried out in triplicate.

## 3 Results and Discussion

### 3.1 Nanoparticle Characterization

H-NMR results showed the successful incorporation of *N*-hydroxysuccinimide (NHS) to the acid-terminated PLGA polymer (Figure 1B) and its subsequent conjugation to NH_2_–PEG–biotin resulting in the PLGA–PEG–biotin copolymer (Figure 1C). The characteristic peaks of PLGA are present in both H-NMR spectra. The signal at 1.5 ppm is related to the CH_3_ group of the lactic acid, and the 4.8 and 5.2 ppm peaks are ascribed to the CH from lactic acid and the CH_2_ from glycolic acid, respectively, in agreement with the previous literature.^22^ The peak at 2.8 ppm in Figure 1B corroborates the activation of the carboxylic acid groups of PLGA, obtaining an NHS-ester derivative used to conjugate the amine–PEG–biotin through a covalent amide bond. Peaks at 1.2 and 3.5 ppm are detected due to the residual diethyl ether used in the synthesis. In Figure 1C, a new peak at 3.6 ppm can be observed, attributed to the CH_2_ moiety of the PEG chain that confirms the formation of the PLGA–PEG copolymer. Biotin peaks are mostly hidden by the polymer chain signals, but two peaks with a weak signal can be detected at 4.3 ppm, confirming the presence of the biotin end-group. The molar amount of PEG in the PLGA–PEG system was estimated from the area under the peaks, and the calculated value was around 14 mol %. We used avidin as a cross-linker to bind the commercially available biotinylated anti-*S. aureus* antibody to the synthesized biotinylated NPs to take advantage of the strong noncovalent interaction between avidin and biotin.

Table 1 compiles the physicochemical characterization of the RIF-loaded and empty NPs in aqueous dispersion at pH = 6.5. As can be seen, the hydrodynamic mean size increases for the empty, nonloaded NPs upon avidin–biotin conjugation due to the incipient agglomeration caused by the cross-linker (e.g., avidin), but ζ potential results showed that the colloidal suspension remains stable (with values between −22 and −50 mV). A supramolecular interaction between avidin (i.e., positively charged) and the NPs (i.e., negatively charged) functionalized with biotin could be responsible for the agglomeration observed, as was previously described in the literature.^23^ However, this agglomeration was reversible under sonication or stirring. Avidin presents four identical subunits (homotetramer), which show high affinity with up to four biotin molecules which could explain the increase in the particle sizes observed. This size increase was not observed for the antibody-functionalized RIF-loaded NPs, probably because the biotinylated antibody present on the surface of the NPs competes for avidin-binding sites reducing the agglomeration. The ζ potential results shown in Table 1 also demonstrate the effective functionalization of NPs resulting in a decrease of the ζ potential value after avidin conjugation that partially neutralized the negative surface charge of the NPs. Through the BCA assay, it was possible to determine that the avidin functionalization efficiency obtained during the synthesis process was 75 ± 5 wt %; once that conjugation was determined, the particles were functionalized with the polyclonal antibody, yielding a result of 64 ± 2 wt %. in terms of functionalization efficiency. Figure 1D–F shows representative SEM images of the PLGA–PEG–biotin NPs and antibody-functionalized NPs along with the size distribution of each type of particle, which agrees with the measurements made by DLS, taking into account that the size of the particles may be slightly larger due to agglomeration caused by the drying process during SEM sample preparation. Again, larger sizes were measured for the NPs after avidin–biotin conjugation (Figure 1E) due to agglomeration because, as we mentioned before, avidin can conjugate up to 4 biotin molecules. This agglomeration was reduced after antibody conjugation by extensive stirring during binding, which rendered monodispersed smaller NPs (Figure 1F).

**Table 1:** Nanoparticle Characterization: Size, ζ Potential (at 6.5 pH), and Polydispersity Index for the Different NPs Prepared are Compiled with the DLS Data^a^

	mean size (nm)	ζ potential (mV)	polydispersity index
PLGA–PEG–biotin	102 ± 2	–51 ± 2	0.22 ± 0.03
PLGA–PEG–biotin–avidin	224 ± 24	–33 ± 1	0.21 ± 0.07
PLGA–PEG-biotin–avidin–antibody	203 ± 19	–30 ± 1	0.20 ± 0.07
PLGA–PEG–biotin–RIF	192 ± 13	–41 ± 2	0.10 ± 0.03
PLGA–PEG–biotin–avidin–RIF	218 ± 19	–24 ± 2	0.22 ± 0.10
PLGA–PEG–biotin–avidin–RIF–antibody	190 ± 17	–22 ± 1	0.21 ± 0.05

a Results are expressed as a mean ± SD of four size and ζ potential measurements.

Figure 1G shows the FTIR spectra of the free antibiotic, loaded, and unloaded particles. A clear signal at 1230 cm^–1^ can be observed in RIF-loaded nanoparticles that are not present in the unloaded ones, confirming the presence of the antibiotic in the particles. This peak would be related to the asymmetric stretching bands of the C–O–C groups in the antibiotic.^26^ The peak shift observed would be related to the chemical interaction between the antibiotic and the nanoparticles, which could be responsible for a controlled release. No new chemical bonds were observed in the FTIR analysis of the RIF-loaded NPs, which could be indicative of supramolecular interactions (i.e., hydrogen bonding and electrostatic interactions) between the RIF and the PLGA nanoparticles. RIF has two p*K*a due to its zwitterion nature (with a p*K*a of 1.7 attributed to the 4-hydroxy and a p*K*a of 7.9 related to the 3-piperazine nitrogen), which implies that at neutral pH, the 3-piperazine nitrogen will provide the molecule with a positive charge which would electrostatically interact with the negatively charged PLGA. The PLGA characteristic vibration bands at 1090–1170 cm^–1^ were also observed, attributed to the C–O–C stretching, and at 1130 cm^–1^ attributed to the rocking vibration of CH_3_.^27^ Characteristic vibration bands for RIF were also detected at 1365 cm^–1^ related to CH_2_ and C=C vibrations, at 1060 cm^–1^ related to −CH, CO, and C–H chemical bonds, and at 987 cm^–1^ (≡C–H, C–H) in agreement with the previous literature.^28^ Despite this interaction, the activity of RIF was preserved after encapsulation, as we corroborated in subsequent antimicrobial efficacy studies.

RIF loading for PLGA–PEG–biotin NPs was 2.6 wt %, while NPs functionalized with the antibody showed a 0.9 wt % RIF DL. There are different factors that influence DL in PLGA derivatives, such as the molar weight, chain structure, and characteristics of the end groups. The low loading capacity of the PLGA–PEG–Biotin–RIF NPs could be due to the fact that the PLGA–PEG system has a high molar mass; therefore, the interactions between the remaining unbound carboxylic groups of PLGA after PEG coupling would show few interactions with the amino groups of RIF, entrapping reduced amounts of the antibiotic.^29,30^ For functionalized NPs, another potential explanation is the loss of RIF during the antibody functionalization step. It is well known that part of entrapped drugs within PLGA matrices remains on the outermost part of the NPs and produces an initial burst release, whereas both matrix erosion and drug diffusion control the release of the remaining entrapped drug providing the construct with sustained release ability. RIF release kinetics (Figure 1H) showed that, before antibody surface functionalization, a rapid burst release was observed, probably attributed to the RIF released from the outmost layer of the NPs. Once the surface was functionalized, a linear release was observed, probably because during the surface antibody functionalization, all of the antibiotic present on the external surface was washed out, and the RIF measured was attributed to the drug diffusion from the NP interior after hydrolysis and erosion of the encapsulating matrix. Despite the reduced DL, the elevated efficacy of the RIF is more than enough to exert high antimicrobial action even at very low concentrations, as we show in the following sections.

### 3.2 Bactericidal Activity

**Figure 2:**
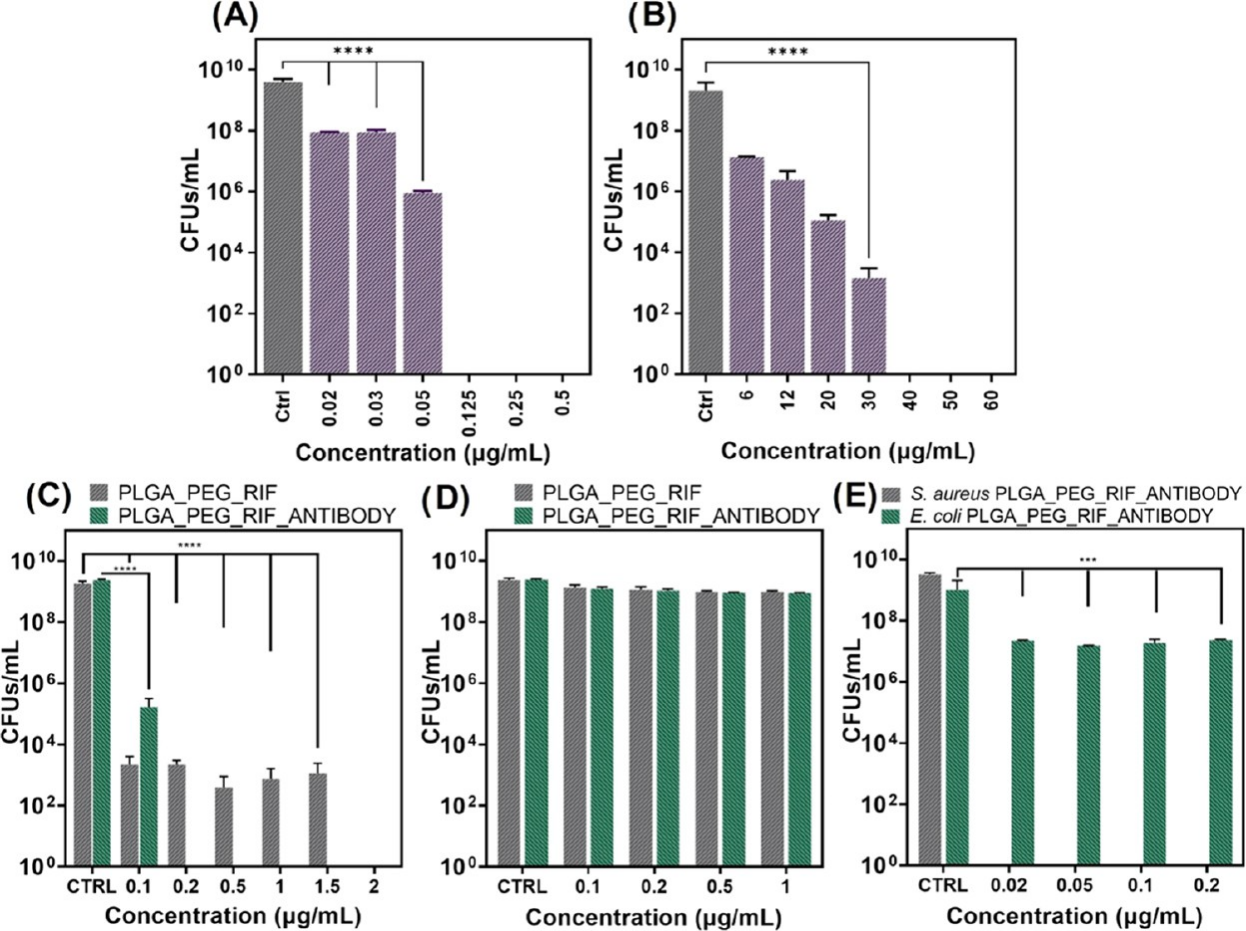
Bactericidal activity of free RIF and RIF-loaded NP treatment in bacterial planktonic cultures: The effect of free RIF in *S. aureus* (A) and in *E. coli* (B) growth. The effect of RIF-loaded NPs with and without antibodies in *S. aureus* (C), in *E. coli* (D), and in the growth of both cocultured strains (E). Bacterial growth is expressed in CFU/mL. Data are depicted as mean ± SD of 4 independent experiments in triplicate (*n* = 12). (*p < 0.05; **p < 0.01; ****p < 0.0001).

The bactericidal effects of the synthesized NPs were evaluated in planktonic cultures of *E. coli*, *S. aureus*, and both bacterial strains together (bacterial coculture), as well as in biofilm models of both bacteria cultured alone and together (mixed biofilm). Figure 2 shows the antimicrobial activity and the in vitro susceptibility tests of equivalent doses of the free and encapsulated RIF against both types of bacterial models.

Free RIF (Figure 2A,B) showed a superior antimicrobial action against Gram-positive bacteria, which is in agreement with the previous literature where it is generally accepted that RIF is bacteriostatic against *E. coli* and bactericidal against *S. aureus*.^31^ MIC and MBC values against *S. aureus* were 0.05 and 0.125 μg/mL, respectively, in agreement with our previous results,^32^ whereas against *E. coli* (Figure 2B), 6 and 40 μg/mL were needed to elicit inhibition and bactericidal action, respectively. The superior antimicrobial action of RIF against Gram-positive bacteria observed is in agreement with other previous reports^33,34^ attributed to its reduced permeability through the outer lipopolysaccharide membrane of Gram-negative bacteria. Considering the MBC/MIC ratio for RIF against both bacterial species, we can define its action as bacteriostatic (i.e., MBC/MIC > 4). The targeted delivery against Gram-positive bacteria is also shown in Figure 2C, where the same RIF-loaded NPs with and without the targeting antibody showed different efficacy. The immunonanoparticles showed an enhanced antimicrobial action by having equivalent doses of the loaded antibiotic, demonstrating the importance of the targeting moiety. At 0.2 μg/mL, complete bacterial eradication was observed for the antibody-functionalized RIF-loaded NPs, whereas only a 5-log reduction was observed for the nontargeted RIF-loaded NPs. As we mentioned before, RIF inhibits DNA-dependent RNA polymerase, which is an enzyme present in the cytoplasm responsible for DNA transcription. The superior efficacy of the immunonanoparticles over the nontargeted ones can be attributed to the improved RIF bacterial internalization and uptake when the NPs were targeted with the anti-*S. aureus* polyclonal antibody, which binds to structural antigens on the surface of the whole bacterium. The selectivity was also corroborated in a coculture model of both bacteria (Figure 2E). At the doses tested, no antimicrobial reduction was observed against *E. coli* (Figure 2D), in agreement with our previous results, but, in the coculture model, the selectivity against *S. aureus* is clearly demonstrated and a large antimicrobial action, compared to the antimicrobial action in the monoculture, was observed due to the additive effect of the interspecies bacterial competition for space and nutrients and the inherent inhibition caused by the antibiotic itself. Some authors have attributed the release of antiadhesion polysaccharides by *E. coli* as the main responsible for the competitive advantage of the Gram-negative bacteria over the positive ones.^35^

**Figure 3:**
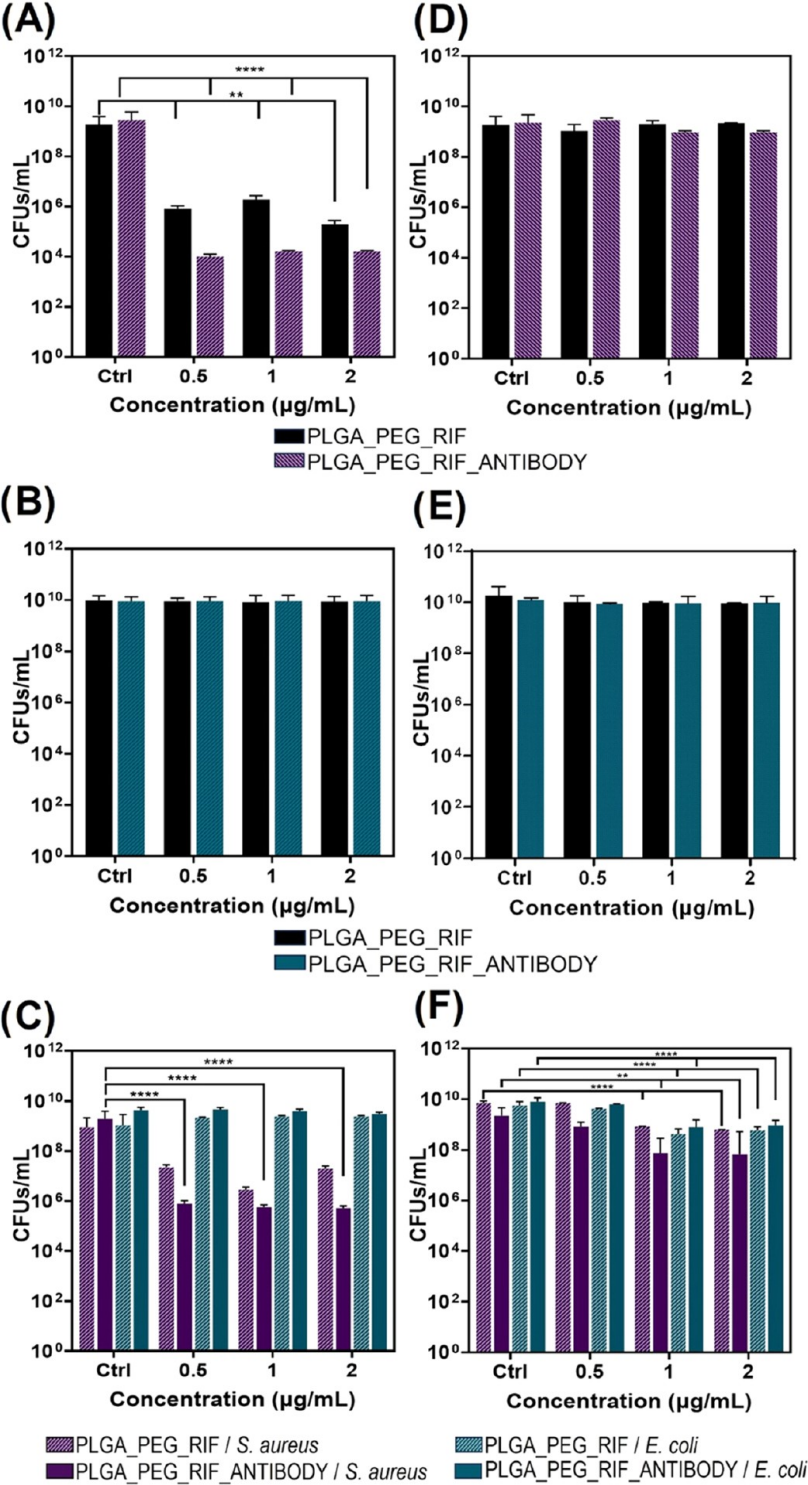
Bactericidal activity of NP treatment in the biofilm: The effect of RIF-loaded NPs with and without antibodies in the inhibition of biofilm formation (A–C) and in the disruption of the already-formed biofilm (D–F). *S. aureus* (A and D), *E. coli* (B and E), and the coculture of both strains (C and F) were used to generate biofilms. Bacterial growth is expressed in CFU/mL. Data are depicted as mean ± SD of 4 independent experiments in triplicate (*n* = 12). (*p < 0.05; **p < 0.01; ****p < 0.0001).

Figure 3 shows the comparative effects of the RIF-loaded immunonanoparticles compared to the nonfunctionalized ones in the inhibition of biofilm formation (Figure 3A–C) or against already-formed mature biofilms (Figure 3D–F). In the prevention of *S. aureus* biofilm formation (Figure 3A), the antibody-functionalized NPs exerted a superior inhibition of bacterial growth (up to 2 logs) compared to the nonfunctionalized ones. However, both types of NPs behaved the same against already-formed mature biofilms (Figure 3D). Again, the targeted nanoparticles can approach the antibiotic more effectively to the bacterial surface than their nontargeted counterparts, and the inhibition of biofilm formation is promoted. The lack of antibiofilm effect against the gram-negative *E. coli* corroborates the results obtained with its planktonic counterparts (Figure 2). Our results are in agreement with previously reported works where similar RIF concentrations reached the minimum biofilm eradication concentration on *S. aureus* strains.^36^ However, once the biofilm is formed, polysaccharides, proteins, and extracellular DNA protect bacteria and hinder antibiotic treatments. Probably, the recognition ability of the polyclonal antibody is highly impaired when diffusing through already-formed mature biofilms. Those negligible effects on biofilm disruption are in agreement with the literature where the doses required to exert antimicrobial action increase orders of magnitude when bacteria are present in their sessile form compared to those required to eliminate them in their planktonic state. For instance, Laverty et al.^37^ showed that against *S. aureus* ATCC 29213, a dose of 0.24 μg/mL was needed to inhibit bacterial growth in their planktonic state, but the minimum biofilm eradication concentration (MBEC) was reached at 15.63 μg/mL, concentration much higher than the highest tested in our work (3 μg/mL). Thill et al.^38^ analyzed 51 clinical strains of RIF-susceptible *S. aureus* and found that 60% of the strains showed an MBC between 0.016 and 0.064 μg/mL, whereas 26% of the strains showed an MBEC above 4 μg/mL. In their study, Reiter et al. also demonstrated that the MIC values for RIF were remarkably low (<0.03 μg/mL) compared to the MBEC (64 μg/mL), which created a statistically significant difference when compared to other antimicrobials tested.^39^ The RIF-loaded antibody-functionalized NPs were capable of reducing the cell counts of *S. aureus* in at least 5-log reduction at a NP concentration of 0.5 μg/mL, but when the concentration of NPs was increased up to 3 μg/mL, there was no considerable improvement in the observed antimicrobial action (Figure 3A). As we mentioned before, at the doses tested, the RIF-loaded antibody-functionalized NPs did not exert any antimicrobial prophylactic effect on *E. coli* biofilm formation and were unable to reduce mature *E. coli* biofilms.

Under coculture conditions, the inhibition of *S. aureus* biofilm formation (Figure 3C) was reduced in the same trend as described in the case of the independent biofilms. The addition of NPs without antibodies inhibited biofilm formation at a lower extent (up to 2 logs) than the antibody-targeted ones. Moreover, the addition of the antibody-targeted NPs to *S. aureus* during biofilm formation exerted a reduction of up to 4 logs at the highest concentration assayed compared to the control samples. Again, no concentration-dependent effect was observed at the concentrations tested. The addition of antibody-functionalized NPs to already-formed coculture biofilms (Figure 3F) showed a very slight reduction of *S. aureus* growth (1-log) compared to the NPs without antibodies. Only a decrease of 2 logs was achieved when antibody-targeted NPs were added at the highest concentrations tested (1 and 2 μg/mL) compared to control samples. As described for the independent biofilms (Figure 3B,E), *E. coli* biofilms did not undergo any change despite the treatment used in the coculture model (Figure 3C,F). These results highlight the successfully targeted activity of the synthesized NPs, which were able to effectively discern between both bacterial strains in the coculture biofilm model.

**Figure 4:**
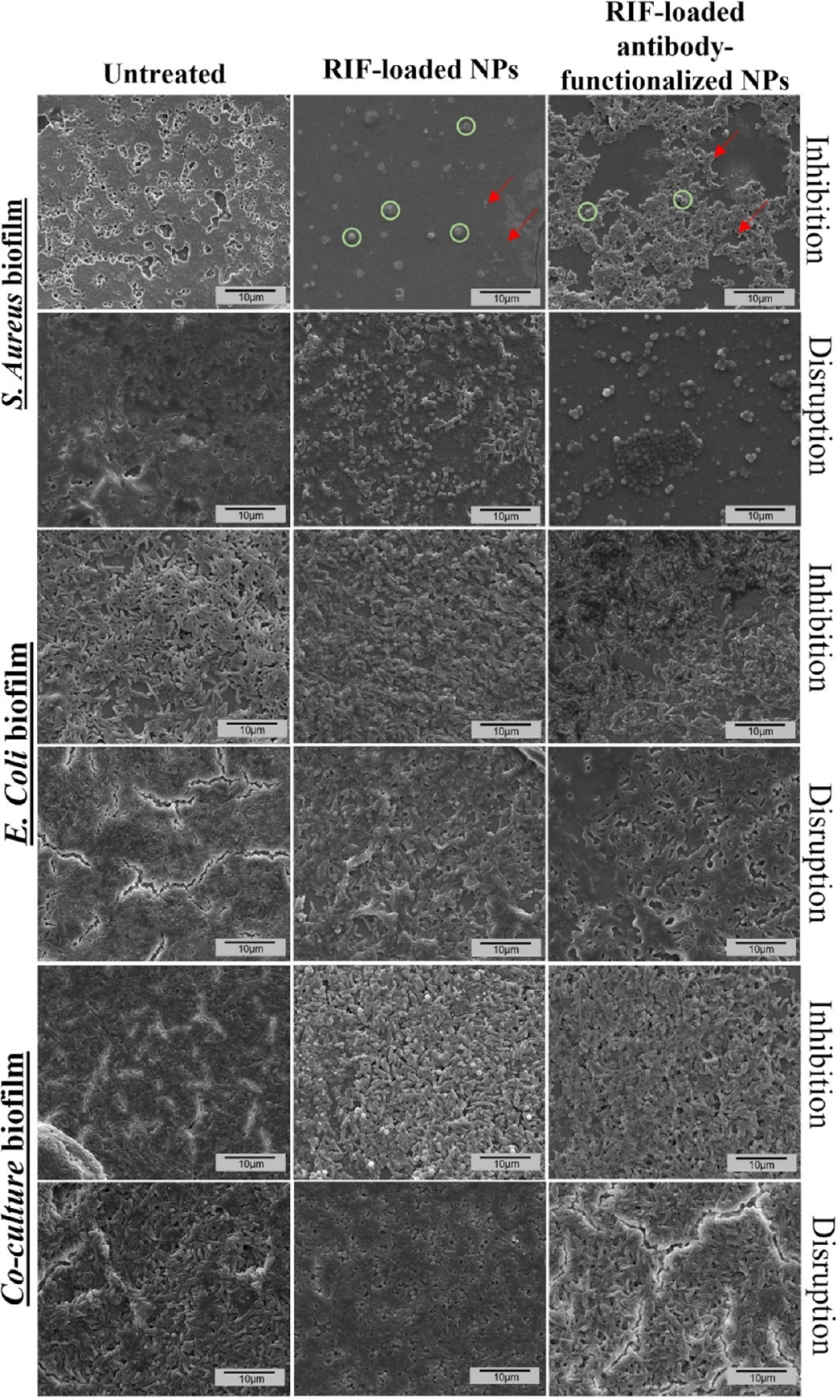
SEM micrographs of bacterial biofilms. *S. aureus*, *E. coli*, and the coculture of both strains were used to generate biofilms. Control samples (not treated biofilms) were also assayed. The effects on biofilms of the RIF-loaded NPs with and without antibodies are depicted. The inhibition of biofilm formation and the disruption of already-formed mature biofilms were analyzed. The aggregation of the particles is indicated in the image by the red arrows, while bacteria are indicated by the green circles in one panel as an example to facilitate visualization. Scale bar 10 μm.

**Figure 5:**
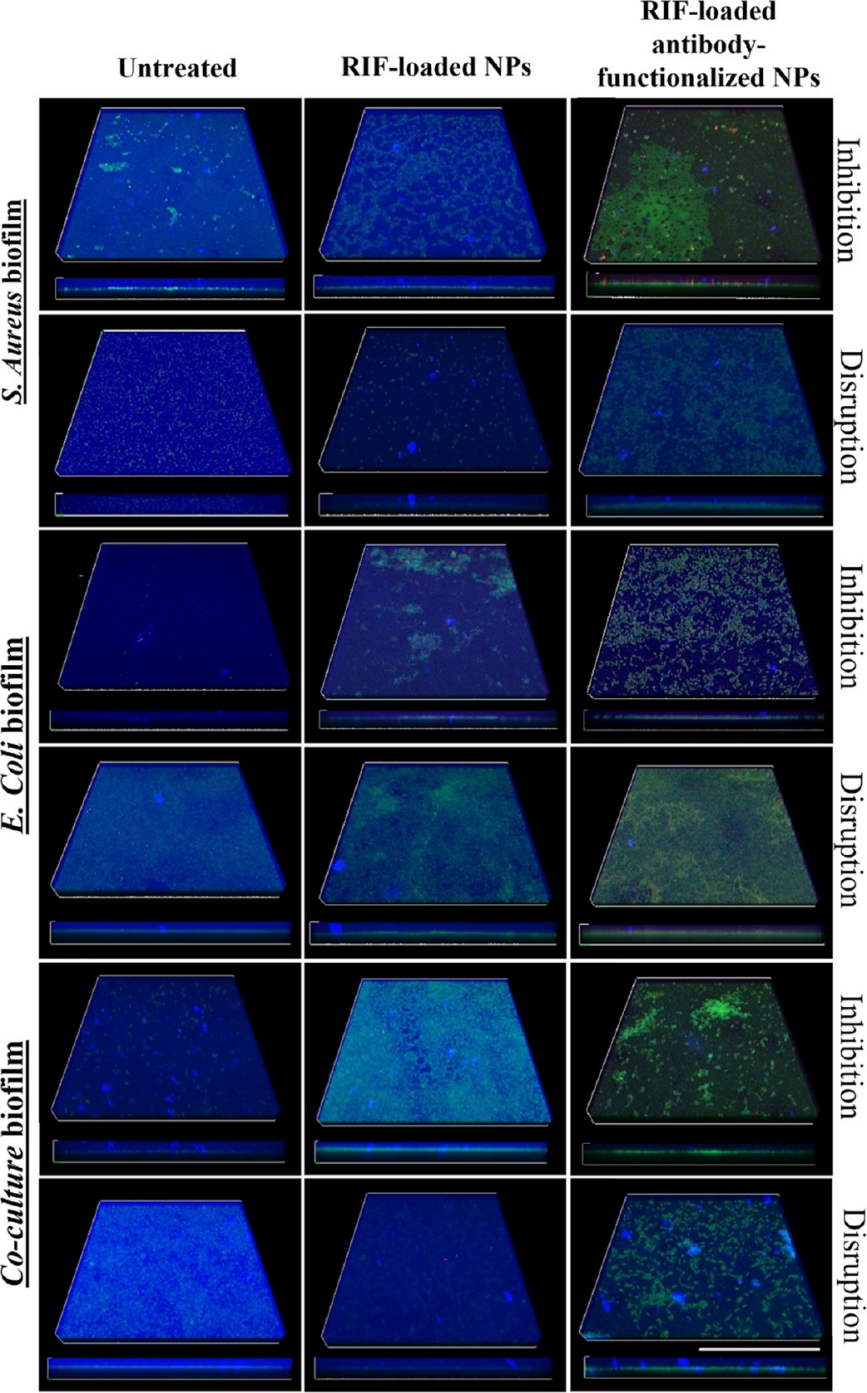
Confocal laser scanning microscopy images of biofilms. *S. aureus*, *E. coli*, and the coculture of both strains were used to generate biofilms. Control samples (not treated biofilms) were also assayed. The effects of the RIF-loaded NPs with and without antibodies on biofilms are depicted. The inhibition of biofilm formation and the disruption of already-formed mature biofilms were evaluated. In all of the samples, bacteria are stained in green by SYTO 9 attached to the bottom of the well, while Calcofluor staining of biofilms is depicted in blue. The bottom images of each sample show each *z*-axis maximum projection image. Scale bar 50 μm.

The morphology of *S. aureus*-based biofilms was also analyzed by SEM. Figure 4 shows the morphology of the nontreated bacteria used as control where the cocci are surrounded by an organic extracellular matrix assigned to the exopolysaccharide matrix (EPS) in agreement with the previous literature.^40^ The effect of the RIF-loaded NPs at inhibitory concentrations (0.5 μg/mL) on *S. aureus*-based biofilms is depicted in the central panel of Figure 4, where a reduction in bacterial growth is observable when compared to the control. However, when using RIF-loaded antibody-functionalized NPs (0.5 μg/mL), the growth was critically reduced, being even able to observe the aggregation of the particles probably caused by the effect of the avidin indicated in the image by the red arrows, while bacteria are indicated by the green circles. Confocal fluorescence microscopy confirms this inhibitory effect, as it can be observed in the confocal images (Figure 5). SYTO 9 stain was used to counterstain bacterial DNA labeling in green both live and dead Gram-positive and Gram-negative bacteria, and calcofluor white was used as a blue-fluorescent dye to bind β-linked polysaccharides such as those present in the bacterial biofilms.^41^ In Figure 5, the control shows a thick EPS top layer (blue) that protects the bacteria present underneath (green) as observed in the orthogonal projection (bottom part of the image), whereas in the *S. aureus* biofilm treated with NPs with and without antibodies, the EPS layer is highly disrupted and isolated bacterial colonies are observed to be more potent when biofilms were treated with antibody-functionalized NPs. These results point again to the specificity of the synthesized NPs and their significant reduction of biofilm formation. The SEM images of preformed biofilms of *S. aureus* depicted in Figure 4 showed a decreased population of bacteria after RIF-loaded NP treatment (0.5 μg/mL) compared to the control sample, even higher when antibody-functionalized NPs were employed. It is possible to consider that EPS disruption could be a potential inhibitory pathway; however, in the confocal images (Figure 5), it can be observed that the reported NPs did not completely disrupt the extracellular matrix when biofilms were treated with RIF-loaded antibody-functionalized NPs, bacteria (both alive and death) are more potent than biofilms, which could involve a reduction in bacterial viability.

Against *E. coli*, the SEM images (Figure 4) revealed uniform bacterial growth, not showing differences between control and treated samples as well as between both treatments (without and with antibodies), which is consistent with the specific targeting of the synthesized NPs against only *S. aureus*. However, in the confocal microscopy imaging, the staining shows that the production of EPS can take up to 48 h, in agreement with the previous literature; therefore, its production is slightly hindered.^42^ It is important to point out that while biofilms of *S. aureus* are strongly attached to the bottom of the wells, *E. coli*, being a motile bacterium, forms biofilms at the air–liquid interface, which makes them difficult to stain. Under coculture conditions, SEM images (Figure 4) showed a significant reduction in *S. aureus* biofilm formation when using antibody-functionalized RIF-loaded NPs (0.5 μg/mL) compared to the control samples and also to those treated with nonfunctionalized NPs. Moreover, some differences can be found between both treatments, as *S. aureus* bacteria were less evident in samples treated with antibody-functionalized NPs. This effect was corroborated in the confocal images (Figure 5), which showed the reduction in the biofilm thickness being more apparent in biofilm samples treated with the antibody-functionalized NPs compared to the nonfunctionalized ones. In addition, differences were also found between the inhibition of biofilm formation vs the disruption of the preformed biofilms, with the treatments being less effective against the latter. To sum up, our promising results underline the efficiency and specificity of the synthesized NPs in the treatment of *S. aureus* biofilms.

### 3.3 Nanoparticle Cytocompatibility and Bactericidal Activity in the In Vitro Infection Model

**Figure 6:**
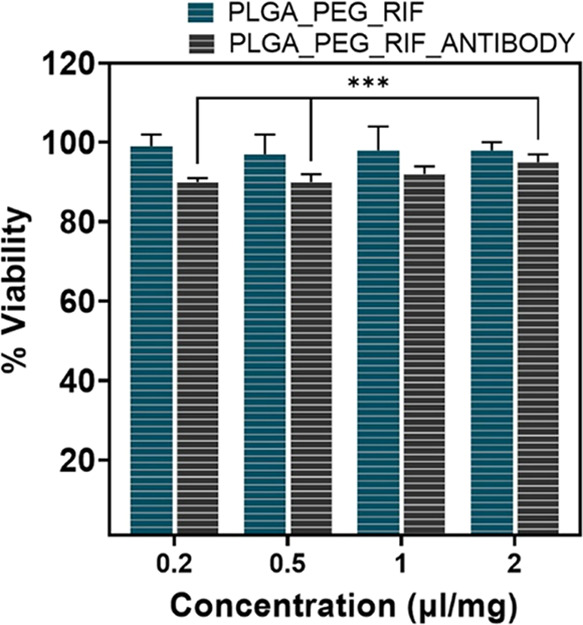
Viability of J774 mouse macrophages after treatment for 24 h with RIF-loaded NPs with and without antibodies. The results (mean ± SD of three replicas) are depicted on the basis of control samples (untreated cells), which were set as 100% viability.

The blue cell viability assay (Figure 6) was carried out to evaluate the in vitro toxicity of RIF-loaded PLGA–PEG NPs with and without the targeting antibody on J774 mouse macrophages after 24 h of incubation. Both types of NPs showed viabilities above 70% at the doses tested, which represents the lowest value recognized by the ISO 10993-5^43^ to consider a material in a medical device as noncytotoxic. For further experiments, we considered targeted and nontargeted NPs at a concentration of 0.5 μg/mL since it was the MBC for planktonic *S. aureus* cultures. It has been demonstrated that this concentration inhibited the *S. aureus* biofilm formation and has been determined to be noncytotoxic. We previously demonstrated that PLGA–PEG NPs show noncytotoxic effects at doses up to 400 μg/mL not only on macrophages but also on fibroblast and keratinocytes.^44^ Several reports also show the noncytotoxic behavior of RIF on murine J774 macrophages (half maximal inhibitory concentration, IC_50_ = 65 μg/mL), corroborating its antibiotic nature.^45,46^

**Figure 7:**
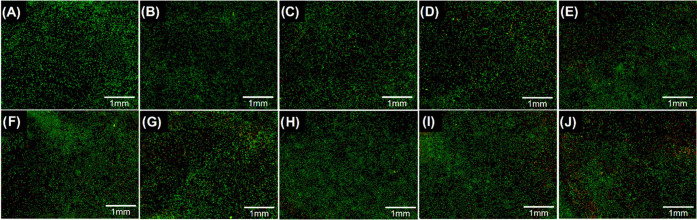
Confocal microscopy analysis of macrophage viability under different experimental conditions. (A) Representative image of uninfected macrophages. Infection of macrophages with *S. aureus* at MOIs of (B) 8:1, (C) 10:1, and (D) 20:1. Infection of macrophages with *E. coli* at MOIs of (E) 3:1, (F) 5:1, and (G) 8:1. Infection of macrophages with a coculture of *S. aureus* and *E. coli* at MOIs of (H) 8:3:1, (I) 10:5:1, and (J) 20:8:1. Magnification 10x.

Cells were infected with *S. aureus* at multiplicities of infection (MOIs) of 8:1, 10:1, and 20:1, while *E. coli* was employed at MOIs of 3:1, 5:1 and 8:1. For the coculture, the same MOIs were combined. The viability of macrophages after infection was also assessed using the live/dead assay and analyzed by confocal microscopy (Figure 7). Overall, the viability of cells at most infective doses was similar to the control sample (Figure 7A). However, at higher doses in the coculture, a very slight decrease in viability was observed. Nevertheless, at these doses, the number of live cells (green staining) remained much higher than the number of dead cells (red staining).

**Figure 8:**
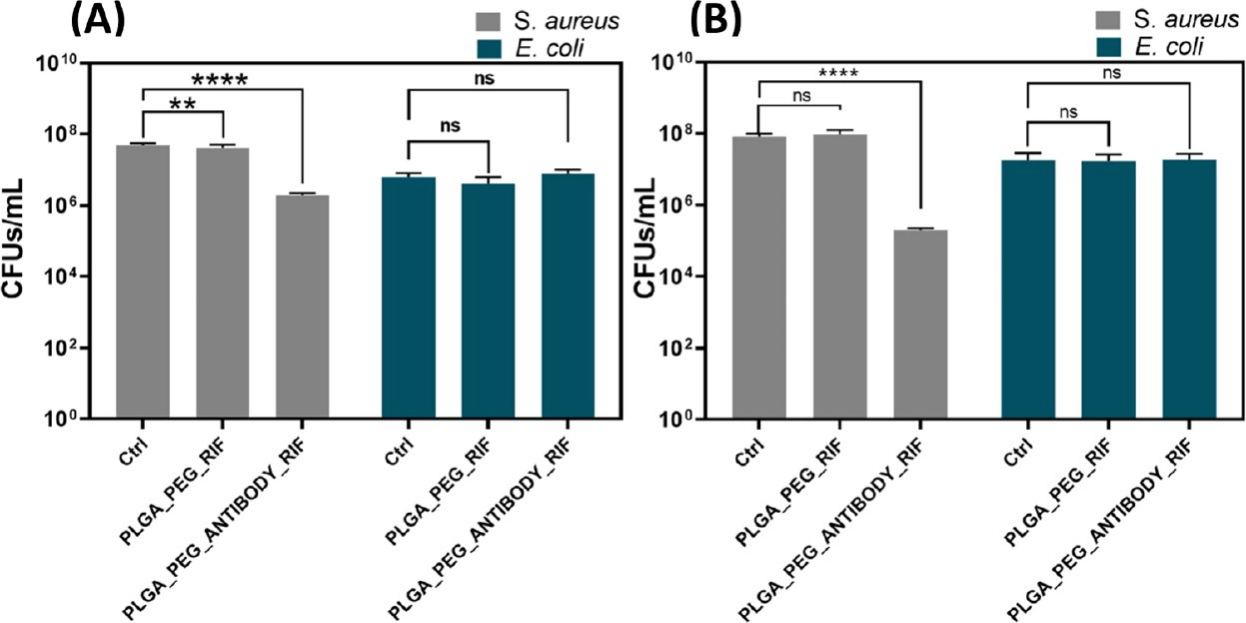
Intracellular bacterial growth in CFU/mL after infection of J774 mouse macrophages with *S. aureus* or *E. coli* alone (A) and with the coculture of both strains (B). Results are depicted as mean ± SD of four independent experiments performed in triplicate (*n* = 12). (*p < 0.05; **p < 0.01; ****p < 0.0001).

The antibacterial activity of RIF-loaded PLGA–PEG NPs surface functionalized with and without the targeting antibody was tested at 0.5 μg/mL concentration (determined in the cytotoxicity study; Figure 8). After treatment, intracellular surviving bacteria were grown on TSA plates and counted after 24 h of incubation with both types of NPs. The bactericidal effects of the selected concentration of NPs were also analyzed in bacterial cultures without macrophages as control, and another control without NPs was also included in the study. The resulting colonies of control bacteria without macrophages did not show any growth, as was expected, because, during the process, gentamicin sulfate was used to eliminate extracellular bacteria, proving that the growth in the bacteria/cell coculture was exclusively attributed to intracellular bacteria. The results of the infection of each strain alone are presented in Figure 8A in terms of bacterial cell counts (CFU/mL). The data indicate that *S. aureus* displayed statistically significant differences in growth when incubated with the RIF-loaded NP surface functionalized with the targeting antibodies in an infection model with only this infective pathogen alone. When incubated with particles lacking antibodies, there were minimal differences compared to the control, suggesting a reduced inhibitory effect of the nanoparticles. In contrast, *E. coli* showed no differences in accordance with the MIC and MBC results, indicating that it did not exhibit any growth variations. In Figure 8B, when both bacteria were incubated in coculture in the infection model, *S. aureus* exhibited a decrease in growth upon contact with the NPs having targeting antibodies, and the difference was statistically significant compared to the nontargeted ones. All in all, we conclude that the targeted nanoparticles showed an enhanced antimicrobial action against intracellular bacteria even when infecting macrophages with both pathogens (*E. coli* and *S. aureus*), with this effect being clearly specific against *S. aureus*.

## 4 Conclusions

Herein, we have demonstrated that targeting bacteria using anti-staphylococcal polyclonal antibodies provides a selective advantage in the elimination of planktonic, sessile, and intracellular infective bacteria. The RIF-loaded antibody-functionalized NPs showed MIC and MBC values against *S. aureus* of 0.05 and 0.125 μg/mL, respectively. Coculture models of *E. coli* and *S. aureus* were also comparatively tested using the immunonanoparticles and the nontargeted ones to represent in vitro part of the polymicrobial nature of different clinical infections. In this scenario, the selectivity against only the Gram-positive bacteria was demonstrated. Those targeted nanoparticles showed an enhanced prophylactic effect on the prevention of biofilm formation though they did not completely eradicate already-formed mature biofilms. The RIF-loaded antibody-functionalized NPs were capable of reducing the cell counts of *S. aureus* single biofilms in at least 5-log reduction at a NP concentration of 0.5 μg/mL. We have also shown that in coculture models of infected eukaryotic cells, murine J774 macrophages, the targeted immunonanoparticles at 0.5 μg/mL reduced in 2-log the prokaryote intracellular infective *S. aureus*. All in all, compared to the use of nontargeted antibiotic-loaded NPs, antibiotic-loaded polymeric NP surface functionalized with targeting antibodies represent a superior approach for the elimination of sessile, planktonic, polymicrobial, and intracellular pathogenic bacteria.
